# Unusual presentation of fibroadenoma in the pelvic region – A case report

**DOI:** 10.1016/j.ijscr.2024.110352

**Published:** 2024-09-26

**Authors:** Adedoyin B. Ojo, Sandra E. Sonusi, Babatunde A. Ayoade, Abeeb B. Oyedele

**Affiliations:** a Department of Surgery, Olabisi Onabanjo University Teaching Hospital, Nigeria; b Department of Morbid Anatomy & Histopathology, Olabisi Onabanjo University Teaching Hospital, Nigeria

**Keywords:** Case report, Ectopic breast tissue, Embryologic milk line

## Abstract

**Introduction and importance:**

Ectopic breast tissue (EBT) may occur anywhere along the milk line, which extends bilaterally from the axilla to the groin. EBT can undergo the same physiologic and pathologic changes seen in normal breast tissues. The most common reported pathologies are carcinoma, inflammatory changes and fibroadenoma.

**Case presentation:**

A painless slow growing swelling on the mons pubis of a young lady which was initially thought to be a lipoma but pathology showed it was ectopic breast tissue is reported.

**Clinical discussion:**

During normal development, mammary ridges form breasts in the pectoral region and their remainder regresses. Failure of regression of the remaining portions of the mammary ridges can lead to ectopic breast tissue development with (polythelia) or without (polymastia) a nipple-areolar complex. Ectopic breast tissue can occur anywhere along the mammary ridge but is most commonly found in the axilla; findings anywhere else outside of the axilla are rare.

**Conclusion:**

A high index of suspicion of ectopic breast tissue is required for swellings over the anatomic distribution of the embryologic milk line particularly if these swellings respond to hormonal changes in puberty, pregnancy and lactation.

## Highlights

• Ectopic breast tissue (EBT) may occur along the milk line, which extends from the axilla to the groin.
• A swelling on the mons pubis of a lady which pathology showed it was ectopic breast tissue is reported.
• A high index of suspicion of ectopic breast tissue is required for swellings over the milk line.

## 1 Introduction and importance

**Fig. 1:**
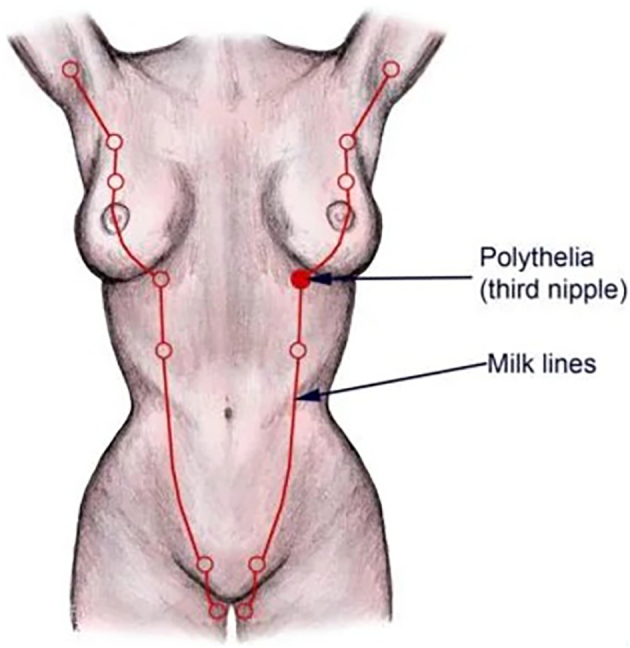
Embryologic milk line.

Ectopic breast tissue (EBT) may occur anywhere along the milk line, which extends bilaterally from the axilla to the groin (Fig. 1) [1]. Fibroadenomata are benign tumors of the breast, so invariably ectopic fibroadenomata may arise in any location along the anatomic distribution of the embryologic milk line [2,3].

The epidemiology of EBT is poorly understood. The frequency of ectopic breast tissue in females is 1 to 6 % and is relatively common in the axilla or the chest wall but is unusual in other sites such as the mons pubis [4]. The presentation of EBT depends on the level of breast tissue developed and its functionality. It tends to manifest during or shortly after pregnancy in response to elevated levels of oestrogen, prolactin, and progesterone [5]. EBT can undergo the same physiologic and pathologic changes seen in normal breast tissues. It can develop benign breast changes such as fibroadenoma and fibrocystic changes. It can also develop malignant changes such as lobular, ductal, mucinous carcinoma and phyllodes [6]. Pathologies developing in an EBT are reported as a rare entity in the literature. The most common reported pathologies are carcinoma, inflammatory changes and fibroadenoma [7].

The wide range of clinical presentations and symptomatology can pose a significant diagnostic challenge, and clinicopathologic correlation is significant in establishing the diagnosis. Although the diagnosis is ultimately made with histologic confirmation, certain clinical characteristics such as the timing of mass enlargement and lactogenesis are important clues [5].

## 2 Case presentation

### 2.1 Gross findings

A young lady presented to our surgical outpatient department with a swelling on her mons pubis of 6 years duration. The swelling was painless and progressively increased in size to approximately the size an egg. There was occasional pain and itching especially during menstruation. She had a 10 cm by 12 cm lobulated, non-tender, soft swelling in the mons pubis with no attachments to the overlying skin or underlying structures on clinical examination. There was no palpable lymphadenopathy. Soft tissue ultrasound scan described a focal largely hypoechoic mass in the mons pubis measuring 8.26 cm × 3.99 cm in dimensions and containing multiple anechoic areas. There was no colour flow on Doppler examination and the mass was not attached to underlying structures. A clinical diagnosis of a lipoma over the mons pubis was initially made. The swelling was excised in the operating room with local anaesthesia and the specimen was sent for pathologic examination.

### 2.2 Pathologic examination

**Fig. 2:**
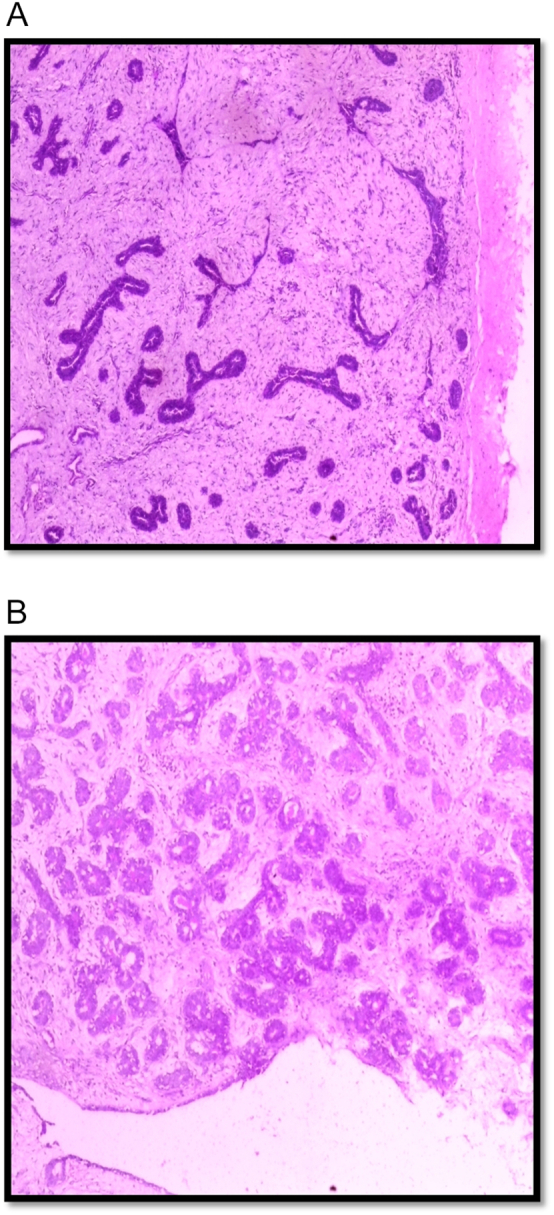
Histologic sections of the mass showing benign biphasic lesion composed of proliferating ducts and stroma (Haematoxylin-eosin staining at ×20 magnification). A, Encapsulated lesion with several compressed ducts in a fibrocollagenous stroma. B, Other areas showing few dilated ducts and adenosis.

Macroscopically, the specimen consisted of a fibro fatty tissue weighing 150 g measuring 9.7 cm × 9.5 cm × 4.8 cm. Serial cut sections revealed homogenous greyish white surfaces. Histologic sections showed a biphasic lesion composed of proliferating fibrocollagenous stroma as well as glandular elements. The ducts are compressed with few that are cystically dilated (Fig. 2A and B). A pathologic diagnosis of fibroadenoma was made.

## 3 Clinical discussion

During the 6th week of embryonic development, breast tissue and glands grow along mammary ridges [2]. These mammary ridges are called “milk lines” and they extend bilaterally from the axilla to the inguinal region [3]. During normal development, mammary ridges form breasts in the pectoral region and their remainder regresses [2,3]. It is postulated that failure of regression of the remaining portions of the mammary ridges can lead to ectopic breast tissue development with (polythelia) or without (polymastia) a nipple-areolar complex. Ectopic breast tissue can occur anywhere along the mammary ridge but is most commonly found in the axilla; findings anywhere else outside of the axilla are rare [2,3]. Patients may not be aware of ectopic breast tissue until later in life as it responds to hormonal changes and may become bigger during puberty, pregnancy, or lactation [2,3].

The frequency of ectopic breast tissue in females is 1 to 6 % and is relatively common in the axilla or the chest wall but is unusual in other areas like the mons pubis [4]. Ectopic breast tissue can undergo physiologic and pathologic changes seen in normal breast tissue such as mastitis, fibrocystic changes, and fibroadenoma [2,3]. The differential diagnosis, in this case, includes fibroadenoma, lipoma, cyst, and although less likely a cutaneous metastasis from an underlying malignancy [3].

Depending on anatomic location, various imaging modalities can be useful in the evaluation of non-specific soft tissue masses [8]. The ultrasound can be used to evaluate the echotexture, and vascularity of a lesion [3]. Sonographic findings of fat lobules and fibroductal tissue resembling normal breast tissue can support the diagnosis of ectopic breast tissue. MRI can also be performed for atypical cases, especially if there is a concern for malignancy to assess the depth of invasion. Mammography should be used for further evaluation when EBT are located in the axilla [8].

Definitive diagnosis is achieved with fine-needle aspiration cytology or biopsy. Most patients with ectopic breast tissue are asymptomatic and therefore do not require treatment if histologic findings are benign. Surgical excision is the treatment of choice for lesions that are symptomatic, have histologic features concerning malignant transformation, or simply in cases where cosmetic removal is desired [8].

This work has been reported in line with the SCARE criteria [9] and it demonstrates a unique case of ectopic fibroadenoma diagnosed in a patient with a swelling in the mons pubis. Pathologic examination assisted immensely in making the diagnosis. Informed consent was given by the patient for publication.

## 4 Conclusion

A high index of suspicion of ectopic breast tissue is required for swellings over the anatomic distribution of the embryologic milk line particularly if these swellings respond to hormonal changes in puberty, pregnancy and lactation.

## Consent

Patient's privacy maintained. Written informed consent was obtained from the patient for publication of this case report and accompanying images. A copy of the written consent is available for review by the Editor-in-Chief of this journal on request.

## Institutional review board approval

Not required.

## Ethical approval

Ethical approval is not required for case reports or case series by the Institutional Review Board of Olabisi Onabanjo University Teaching Hospital, Nigeria.

## Funding

None.

## Author contribution

ABO - Adedoyin B. Ojo: Concepts, Design, Definition of intellectual content, Literature search, Clinical studies, Data acquisition, Manuscript preparation, Manuscript editing, Manuscript review, Guarantor.

SES - Sandra E. Sonusi: Definition of intellectual content, Clinical studies, Manuscript preparation, Manuscript editing, Manuscript review.

BAA - Babatunde A. Ayoade: Definition of intellectual content, Manuscript preparation, Manuscript editing, Manuscript review.

ABO - Abeeb B. Oyedele: Definition of intellectual content, Literature search, Clinical studies, Data acquisition, Manuscript preparation, Manuscript editing, Manuscript review.

## Guarantor

Adedoyin B. Ojo.

## Research registration number

Not applicable.

## Conflict of interest statement

None.
